# Capacity for ethical and regulatory review of herbal trials in developing countries: a case study of Moringa oleifera research in HIV-infected patients

**DOI:** 10.1186/s40545-017-0099-5

**Published:** 2017-02-20

**Authors:** Tsitsi G. Monera-Penduka, Charles C. Maponga, Gene D. Morse, Charles F. B. Nhachi

**Affiliations:** 1 Drug and Toxicology Information Services (DaTIS), School of Pharmacy, University of Zimbabwe College of Health Sciences, Harare, Zimbabwe; 2 Center for Integrated Global Biomedical Sciences, University at Buffalo, Buffalo, New York USA; 3 Department of Clinical Pharmacology, University of Zimbabwe College of Health Sciences, Harare, Zimbabwe

**Keywords:** Herbal trial, Regulation, Developing countries

## Abstract

**Background:**

Lack of regulatory capacity limits the conduct of ethical and rigorous trials of herbal medicines in developing countries. Sharing ethical and regulatory experiences of successful herbal trials may accelerate the field while assuring human subjects protection. The methods and timelines for the ethical and regulatory review processes for the first drug regulatory authority approved herbal trial in Zimbabwe are described in this report.

**Methods:**

The national drug regulatory authority and ethics committee were engaged for pre-submission discussions. Six applications were submitted. Application procedures and communications with the various regulatory and ethics review boards were reviewed. Key issues raised and timelines for communications were summarized.

**Results:**

There was no special framework for the approval of herbal trials. One local institutional review committee granted an exemption. Key issues raised for revision were around pre-clinical efficacy and safety data, standardization and quality assurance of the intervention as well as consenting procedures. Approval timelines ranged between eight and 72 weeks.

**Conclusions:**

In the absence of a defined framework for review of herbal trials, approval processes can be delayed. Dialogue between researchers and regulators is important for successful and efficient protocol approval for herbal trials in developing countries.

**Trial registration:**

The study was registered prospectively on August 3, 2011 with clinicaltrials.gov (NCT01410058).

## Background

Clinical trials are considered the gold standard in terms of evidence of efficacy and safety of herbal medicine, yet very few trials are conducted in developing countries. Insufficient capacity, including establishment of ethical and regulatory frameworks to support the conduct of herbal trials are often cited as a contributing factor [1, 2].

Current strategies to enhance capacity of researchers in developing countries to conduct ethical and rigorous herbal trials are focused on research training and resource mobilization and have not included sharing experiences involved in successful ethical and regulatory review processes [2, 3].

Clinical studies of herbal medicines should be designed to produce new, beneficial and generalizable knowledge that will improve health outcomes while assuring human subjects protection. However, difficulties in determining shared concepts of social value, scientific validity and favorable risk–benefit ratio, make this challenging. Nevertheless, herbal medicine research must endeavor to achieve a balance between scientific validity and ethics [4]. Public dissemination of ethical and regulatory processes, issues and challenges by researchers involved in herbal trials in developing countries will provide a platform to learn and build capacity for other researchers. In January 2013, the Zimbabwean national drug regulatory authority approved the first trial with a herbal intervention; the Moringa oleifera supplementation by patients on antiretroviral therapy (MOART) study. In this article we describe the ethical and regulatory processes that were involved; including the human subject protection issues raised and the time taken to acquire the requisite approvals. This experience should stimulate discussion and facilitate the set up of regulatory and ethical frameworks to support the conduct of herbal trials in other developing countries.

## Methods

### Description of the herbal trial

The MOART study was conducted by scientists from the University of Zimbabwe to determine if antiretroviral drugs are affected by concurrent intake of *Moringa oleifera* Lam, a frequently taken dietary supplement in Zimbabwe. This is important because herbal medicines may interact with conventional medicine resulting in increased or decreased drug exposure. The drugs nevirapine and efavirenz were studied. Both drugs are routinely used as part of combination therapy for treating HIV. The study was an intensive pharmacokinetic sampling study which compared pre- and post *Moringa oleifera* Lam. plasma concentration profiles of efavirenz and nevirapine in HIV-infected people on antiretroviral therapy. Moringa was administered as a standardized dry leaf powder in a one sequence cross-over, open label, phase I/IIa, observational study that was conducted over 35 days. Venous whole blood and urine were sampled after a 21-day washout period as well as on day 35 following 14 days of moringa supplementation. Compliance to medication and supplementation restrictions was assessed through reported adherence, pill counts and evaluation of a food/herb/medication diary. The trial is registered with clinicaltrials.gov NCT01410058.

### Ethical and regulatory framework

In Zimbabwe, there are three regulatory bodies involved in clinical research, namely the Medicines Control Authority of Zimbabwe (MCAZ), the Medical Research Council of Zimbabwe (MRCZ) and the Research Council of Zimbabwe (RCZ). MCAZ is the national drug regulatory (NDRA) authority which approves and monitors all clinical trials in Zimbabwe in terms of Part III of the Medicines and Allied Substances Control Act [Chapter 15:03]. It has a well-established framework encompassing trial approval procedures and conduct guidelines, reporting standards and access to clinical trial and pharmacovigillence experts.

Before an application for the conduct of a conventional trial can be submitted to the NDRA, the protocol needs to go through ethical review by the ethics committees of the institutions responsible for the trial (IERC). Thereafter, parallel submissions to the the national ethics committee (NEC) and the NDRA may be made. The MRCZ is the NEC. It was established by the Government of Zimbabwe Notice No. 225 of 1974 and approves and provides ethical oversight for all health research.

When the protocol has a foreign co-investigator or planned bio-specimen shipment outside Zimbabwe, additional review and approval by the national research council (NRC) is required. The RCZ is the NRC. It is a statutory body established by the Research Act [Chapter 10:22] which promotes research in all fields and coordinates foreign/and or foreign funded research. All applications involving health research made to the NRC must go through the MRCZ.

The MCAZ, the MRCZ and the RCZ have publicly available guidelines based on the Declaration of Helsinki, the Council of International Organizations of the Medical Sciences (CIOMS) Guidelines as well as the International Conference on Harmonization for Good Clinical Practice guidelines.

### Herbal protocol review procedures

At the onset, we engaged the MCAZ and the MRCZ in pre-submission discussions and established that there was no special framework for the approval of herbal trials. We were advised to work within the framework for conventional trials.

Following the conventional trial framework, four ethical and regulatory approvals were required for the MOART trial. First, ethical approval was sought from the principal investigator’s institution. Upon approval by the IERCs, further applications were made to the national ethics committee, followed by the national drug regulatory authority. Due to unplanned bio-specimen shipment necessitated by limited capacity for one of the analyses, bio-specimen shipment approval also had to be sought from the NRC.

During the course of the applications the funding collaborators also indicated that their institutions required the protocol to be submitted to them for ethical review. Subsequently, two further ethical review applications were submitted to the institution of the foreign collaborating funder; as well as the institution of a local collaborating funder, bringing the total number of ethical and regulatory review submissions to six. However, the IERC for the local collaborator granted an exemption for ethics review. The exemption was based on the fact that another local IERC (IERC1) had already granted approval and the NEC was also reviewing the protocol.

The local regulatory and ethics committees reviewed a draft of the manuscript for accuracy.

## Results and discussion

### Feedback from the presubmission discussions

During the presubmission discussions the NDRA and NEC personnel directed the researchers to the complete set of forms and submissions required for the conventional trial application. Guidance was sought on how to best adapt our responses to fields on the forms that were originally intended for conventional trials. Discussions regarding fields related to the trial (herbal) intervention as well as existing preliminary data were particularly useful in marrying our planned approach to that of the regulators. This improved the efficiency with which the application package was submitted.

### Further information requested to support initial applications

The researchers were requested to provide additional information on several aspects by the 5 bodies (Table 1). Some issues were raised as recommendations to improve understanding of the study documents while others were required as corrections. The three ethics committees recommended that more detailed descriptions of study procedures be given in both the protocol and the informed consent document. In addition, the IREC2 required a more detailed plan for adverse events monitoring and reporting. Both the requests were straightforward and constituted standard clarification and detail issues applicable in any clinical trial protocol. The initial protocol therefore was not complete and the oversight of the ethics committee was valuable. The missing details were all addressed satisfactorily in the first responses.

**Table 1:** Further information requested to support initial applications

Domain	IERC 1	IERC 2	NDRA	NEC	NRC
Details of research procedures	X	X	X	X	
Details of informed/storage consent	X	X		X	
Reimbursement		x	x		
Details of research design		X	X		
Pre-clinical data to justify human studies			X		
Details of adverse event monitoring		X			
Details of product standardization and quality		X	X		
Adequacy of clinical trial site infrastructure			X		
Re-consenting of participants for specimen shipment					X

In addition, the NDRA, NRC and the IERC2 required justification and corrections around pre-clinical efficacy and safety data, standardization and quality assurance of the intervention as well as consenting procedures. These requests constitute the more challenging issues around scientific validity of research, risk-benefit assessments and informed consent that are commonly cited as core considerations for herbal trial protocols [4, 5]. Regarding preclinical data, the issue raised was that there were no published studies assessing the effect of moringa on efavirenz or nevirapine whose positive findings would typically inform the need for a human study. In response, reference was made to two World Health Organization (WHO) guidelines for research on traditional medicine, which recommend a rapid response research mechanism for herbs. The guidelines cite prior voluntary use of herbal medicines and documented experience of long-term use without evidence of safety problems as is the case with *Moringa oleifera,* as a basis of the risk assessment [6, 7]. Additional preclinical studies assessing the effect of Moringa on metabolic pathways shared by efavirenz and nevirapine with positive findings were also added to the protocol to augment the safety data.

In the initial application, we had proposed to use a single batch of Moringa from the most commonly cited local supplier to maintain generalizability of the findings. The NDRA however highlighted the need to use *Moringa oleifera* of verified and reproducible quality and required that measures be put in place and stated explicitly that would guarantee the integrity of the intervention. Previous herbal medicine reviews have cited product quality and standardization as a common challenge, particularly in light of the need to preserve external validity [4, 8]. At the time of the application, the NDRA did not regulate herbal medicine and did not cite any specific quality criteria to address the quality issue. Following the WHO Guidelines for assessing herbal medicines we assessed three different batches of moringa from the local market for microbial contamination and heavy metal content. Unfortunately, all three failed to meet the WHO criteria [9]. We then proposed an alternative approach which involved processing a batch of moringa leaf powder using good manufacturing practice (GMP) principles specifically for the trial, which would then be externally assessed for quality before proceeding with the trial. This was acceptable to the NDRA. The batch was subsequently processed by the School of Pharmacy and externally assessed for microbial and heavy metal contamination in the Departments of Biological Sciences and Chemistry laboratories respectively.

At the time of protocol approval all analysis was planned locally and as a result the consent form did not cover aspects of specimen shipment. When application was made to the NRC for bio specimen shipment approval, the NEC required additional consent from participants with the affected analysis before approval could be granted. This was done through targeted contact at routine HIV clinic visits and support group meetings.

### Divergent requirements

The IERC2 required the use of their ‘standard statement for reimbursement’ for consent which stated that, *“In the unlikely event that you become ill or injured as a direct result of participating in this study, you understand that you may receive medical care, but it will not be free of charge even if the injury is a direct result of my participation”*. This request was not necessarily unique to herbal trials but comes up often in collaborative trials. It becomes important to discuss in this context because many herbal trials in developing countries will be collaborative with developed countries coming in as the funders [10].

While the statement was consistent with consent documents for other studies conducted in the United States of America [11], it was not compliant with the Helsinki declaration and CIOMS. It was also not in line with the NEC recommendation, which states *“In the event of injury resulting from your participation in this study, treatment shall be offered by the study”*. Globally, there is no consensus on the issue of compensation for research related injuries [12]. One school of thought says that because a participant has accepted that there may be risks and harms that can occur as a part of the study, they should not be compensated in case of an injury [13]. The other says that the consent process cannot be extrapolated to argue that the participant also consents to be harmed during the trial, as risk and harm are often unknown and unexpected and should be compensated [13, 14]. In response we highlighted the differences in health insurance frameworks in the local context that made the NEC require free medical treatment or participant insurance for all research participants. In addition, we highlighted that the requirements of the ethics committee of the study setting should take precedence over the foreign collaborator ones. The response was acceptable to the IERC2. In fact, current versions of their ‘standard statement for reimbursement’ now incorporate some exemptions for covering costs for research-related injurues. Our experience supports previous publications that have recommended democratic deliberations between researchers and regulators to resolve challenges in herbal medicine research [4].

### Approval timelines

**Fig. 1:**
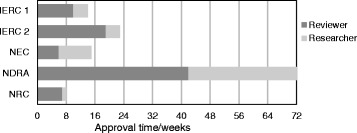
Time taken to obtain approvals. The *black bars* represent the time taken by the reviewer, while the *grey bars* represent the researcher’s response time

Figure 1 shows the distribution of the time taken to get approval between researchers and reviewers. The data incorporates weekends and public holidays.

Delay in ethical and regulatory approval extends the duration of a trial, thereby increasing its cost and potentially risk losing relevance. This often causes tension between researchers and ethics committees [15]. The NEC review had the shortest review committee turnaround time. Ethics committee turnaround time is usually implicated in protocol approval delays [15]; contrary to the current experience in the NEC time was shorter that the respective researcher time. This could be attributed to it being the only committee that had published turnaround times for review of applications, indicating that they have put some thought and effort into improving their efficiency.

The deliberations with the NDRA however took over a year. The NDRA had the responsibility to thoroughly assess existing evidence and quality assurance. The apparent delay may have therefore have improved the protection of participants. Delays in meeting review timelines maybe justified complex protocols where there is need to study the application in-depth, or to get additional essential information from the applicant and to avoid unjustified risks. The distribution in the response time between the researchers and the regulators was 59:41% indicating that the issues raised demanded significant processing time from both reviewers and researchers. For instance, the issue of guaranteeing product quality required assessing several possible options before reverting to the NDRA. This mirrors the current global debate on the complex regulation issues associated with trials of herbal medicines [4, 8].

At the end of 2016, the Ministry of Health and Child Care in Zimbabwe set up a Complementary and Traditional Medicine Research Committee that would going forward, sit to review traditional medicine research including clinical trials. The move will go long way to promote successful and efficient protocol approval for future herbal trials in Zimbabwe.

Some time was also lost due to slow administrative procedures such as delays in delivery responses of interim communications or notification of researchers to collect the same. The results should be interpreted in light of the limitation that the data is from one study, being the only clinical trial of a herbal medicine approved by the NDRA.

### Recommendations

There is a need to develop some guidance for the ethical and regulatory review of clinical trials with herbal interventions. The WHO guidelines on research on traditional medicine could be used as a reference to develop an adapted national regulation. This is particularly important for aspects of clinical trials that differ between “modern” and “traditional” medicines, such as pre-clinical data, standardization and quality assurance of the intervention.

Pre-submission discussions to clarify correct application procedures and identification of key areas can facilitate efficient and successful reviews.

Open and transparent engagement between researchers, ethics committees and regulators during the application process can facilitate resolution of ethical challenges in herbal trial protocols. This is applicable to deliberations between researchers and local as well as international regulators.

Publication of target turn around times for the different type of applications is a strategy that could be employed by other regulatory and ethics review bodies to monitor their efficiency and assist with researchers planning.

## Conclusion

As far as can be ascertained, this report is the first to describe the ethical and regulatory approval processes of setting up a herbal clinical trial in a developing country. The experiences highlight the importance of dialogue between researchers and regulators for successful and efficient protocol approval for herbal trials in developing countries.

## Abbreviations

IERC: Institutional ethics review committee
IERC1: Local institutional ethics review committee
IERC2: Foreign collaborating institutional ethics review committee
MCAZ: Medicines Control Authority of Zimbabwe
MOART: Moringa oleifera supplementation by patients on antiretroviral therapy
MRCZ: Medical Research Council of Zimbabwe
NDRA: National drug regulatory authority
NEC: National ethics committee
NRC: National research council
RCZ: Research Council of Zimbabwe
WHO: World Health Organization
